# Supplementary material for: How do caves breathe: The airflow patterns in karst underground
Source: PLoS One. 2023 Apr 3;18(4):e0283767. doi: 10.1371/journal.pone.0283767 (PMC10069778; doi:10.1371/journal.pone.0283767)
Supplement: S1 Appendix — See the appendix for a derivation of the analytical approximation for a driving pressure in a general double-slope passage with an exponential temperature profile. (PDF) [file pone.0283767.s001.pdf]

Supporting information S1: An analytical approximation for a driving pressure

To get more general insights, lets now calculate an approximation for the driving pressure in a double-slope passage (Fig S1) with an exponential temperature profile of the form,

$$T(x) = T_{in}(1 - \delta T e^{-x/\lambda}), \tag{S1}$$

where $\delta T = (T_{in} - T_{out})/T_{in} = \Delta T/T_{in}$. The elevation difference is $\Delta z = \Delta z_1 + \Delta z_2$, while α_i is the slope angle of each segment. Note that slope angles α_i or Δz_i can also be negative for V-shape or Λ-shape profile; L is the length of the passage, and knickpoint at $x = kL$. The airflow direction must be assumed in advance; we assume updraft during the cold period ($\Delta T > 0$) and downdraft during the warm period ($\Delta T < 0$).

To calculate the driving pressure, we follow the loops shown in Fig S1. For airflow direction $E_1 \to E_2$ (blue loop) we first calculate the pressure change along the passage and then return to z_1 along the external atmosphere, which is here taken as isothermal. The resulting driving pressure is $p_1^o - p_1$. For airflow direction $E_2 \to E_1$ (red loop), we follow the flow along the passage from z_2 to z_1 and then outside back to z_2, resulting in $\Delta p = p_2^o - p_2$.

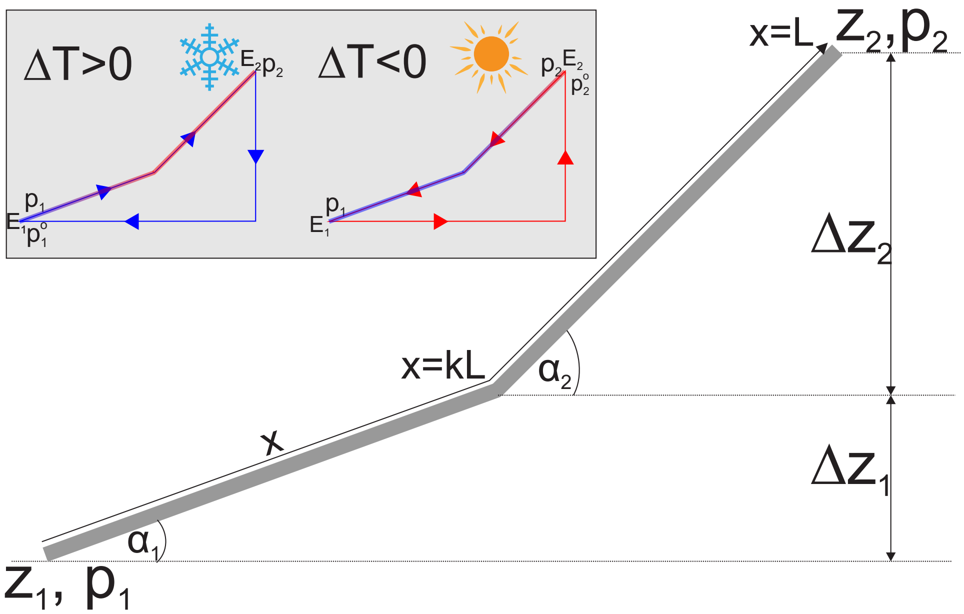

Fig S1. The concept and parameters used for analytical approximation.

Pressure variation along the blue line in the passage is given by:

$$dp = -\rho(x)dh(x) = -\frac{pMg}{RT(x)} \sin\alpha(x)dx. \tag{S2}$$

Since the temperature is a function of distance from the entrance x, we keep the x-coordinate as a master variable. The elevation change within dx is $\sin\alpha(x)dx$. We reorder and integrate Eq S2 along the two

passage segments:

$$\int_{p_1}^{p_2} \frac{dp}{p} = -\int_0^L \frac{Mg}{RT(x)} \sin\alpha(x) dx =$$
$$= -\frac{\sin\alpha_1}{z_{in}} \int_0^{kL} \frac{dx}{1-\delta T e^{-x/\lambda}} dx - \frac{\sin\alpha_2}{z_{in}} \int_{kL}^{L} \frac{dx}{1-\delta T e^{-x/\lambda}} dx \tag{S3}$$

where z_{in} and z_{out} are characteristic length-scales for pressure variation in the isothermal atmosphere with T_{in} and T_{out}, $z_{in} = RT_{in}/Mg$ and $z_{out} = RT_{out}/Mg$. Integration and some algebra give:

$$p_2 = p_1 e^{-\Delta z/z_{in}} \left(\frac{1-\delta T e^{-kL/\lambda}}{1-\delta T}\right)^{-\frac{\lambda \sin\alpha_1}{z_{in}}} \left(\frac{1-\delta T e^{-L/\lambda}}{1-\delta T e^{-kL/\lambda}}\right)^{-\frac{\lambda \sin\alpha_2}{z_{in}}} \tag{S4}$$

$$p_1^o = p_2 e^{\Delta z/z_{out}}, \tag{S5}$$

The expressions in brackets of Eq S4 are the ratio of air temperatures $T(kL)/T_{out}$ and $T(L)/T(kL)$. An expression for the driving pressure becomes:

$$\Delta p_\uparrow = p_1^0 - p_1 = \tag{S6}$$
$$= p_1 \left(e^{\Delta z(\frac{1}{z_{out}} - \frac{1}{z_{in}})} \left(\frac{T(kL)}{T_{out}}\right)^{-\frac{\lambda \sin\alpha_1}{z_{in}}} \left(\frac{T(L)}{T(kL}\right)^{-\frac{\lambda \sin\alpha_2}{z_{in}}} - 1 \right)$$

We do the same for downdraft by following the red loop in Fig S1 to get:

$$\Delta p_\downarrow = p_2^o - p_2 = \tag{S7}$$
$$= p_2 \left(e^{\Delta z(\frac{1}{z_{in}} - \frac{1}{z_{out}})} \left(\frac{T((1-k)L)}{T_{out}}\right)^{\frac{\lambda \sin\alpha_2}{z_{in}}} \left(\frac{T(L)}{T((1-k)L)}\right)^{\frac{\lambda \sin\alpha_1}{z_{in}}} - 1 \right)$$

For cases where the exponents in Eq S6 and EqS7 are small, we take the first-order approximations for exponential and power functions and keep first-order terms in the resulting products. Eq S6 and Eq S7 then become:

$$\frac{\Delta p_\uparrow}{p_1} = \Delta z \left(\frac{1}{z_{out}} - \frac{1}{z_{in}}\right) - \frac{\lambda}{kL} \frac{\Delta z_1}{z_{in}} \ln\frac{T(kL)}{T_{out}} - \frac{\lambda}{(1-k)L} \frac{\Delta z_2}{z_{in}} \ln\frac{T(L)}{T(kL)} \tag{S8}$$

$$\frac{\Delta p_\downarrow}{p_2} = \Delta z \left(\frac{1}{z_{in}} - \frac{1}{z_{out}}\right) + \frac{\lambda}{(1-k)L} \frac{\Delta z_2}{z_{in}} \ln\frac{T((1-k)L)}{T_{out}} +$$
$$+ \frac{\lambda}{kL} \frac{\Delta z_1}{z_{in}} \ln\frac{T(L)}{T((1-k)L)} \tag{S9}$$

We used trigonometric relations between slope angles, segment lengths and elevation change (see Fig S1). The first term in Eq S8 and Eq S9 presents the driving pressure for an isothermal passage with temperature T_{in}. The other two terms are corrections due to the temperature profile. These terms reduce the maximal

isothermal pressure if Δz_1 and Δz_2 are positive: In cold periods, the air temperature along the passage increases, therefore, $T(kL)/T_{out} \geq 1$ and $T(L)/T(kL) \geq 1$ and their logarithm higher than zero. The opposite is valid for the downdraft in the warm period.

In a V-shape scenario with $\Delta z_2 = \Delta z_V$, $\Delta z_1 = -\Delta z_V$ and $k = 0.5$, the driving pressure becomes:

$$\frac{\Delta p}{p_1} = 2(\lambda/L)(\Delta z_V/z_{in}) \ln \frac{T(kL)^2}{T(L)T_{out}} \tag{S10}$$

For $T(L/2) \approx T(L) \approx T_{in}$, the argument of the logarithm becomes T_{in}/T_{out}, the result also show the linear relation between Δz_V and Δp, and therefore square root relation between Δz_V and airflow velocity.

For an L-shape scenario with $\Delta z_1 = 0$, we get

$$\frac{\Delta p_\uparrow}{p_1} = \Delta z \left(\frac{1}{z_{out}} - \frac{1}{z_{in}} \right) - \frac{\lambda}{kL} \frac{\Delta z}{z_{in}} \ln \frac{T(L)}{T(kL)}, \tag{S11}$$

$$\frac{\Delta p_\downarrow}{p_1} = \Delta z \left(\frac{1}{z_{in}} - \frac{1}{z_{out}} \right) + \frac{\lambda}{(1-k)L} \frac{\Delta z}{z_{in}} \ln \frac{T(1-kL)}{T_{out}}. \tag{S12}$$

It is likely that the air is in thermal equilibrium with the massif at the knickpoint ($kL \gg \lambda$ and $(1-kL) \gg \lambda$). In this case $T(L)/T(kL) \approx 1$ and the second term for $\Delta p_\uparrow$ becomes 0, so the updraft is driven by a full "isothermal" pressure. During downdraft, the argument of the logarithm is close to (T_{in}/T_{out}), which considerably reduces the $\Delta p_\downarrow$.

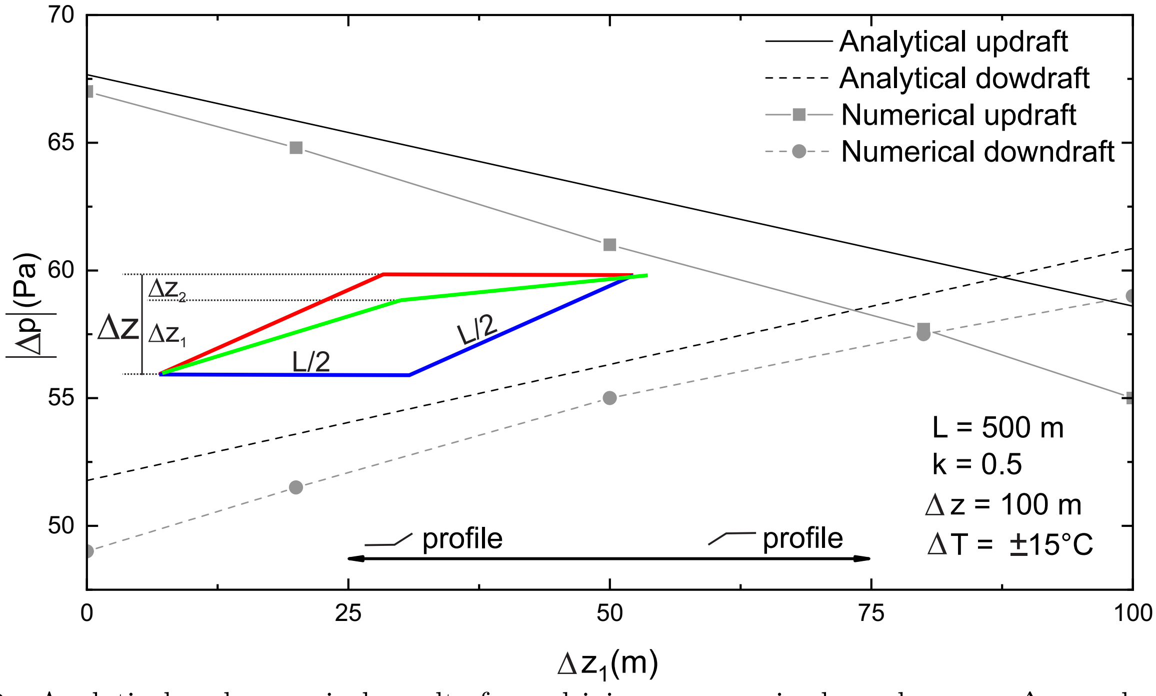

Fig S2. Analytical and numerical results for a driving pressure in dependence on Δz_1 and constant $\Delta z = 100m$, $L = 500m$, $D = 2m, \lambda = 34m$ and $k = 0.5$.

The results provide a basis for understanding the airflow pattern and seasonal airflow asymmetry in a

double-slope passage. Relaxation length λ is given by $\lambda \approx 15D^{1.2}v^{0.2}$, where the velocity is *a priori* not known, but week dependence of λ on the velocity allows a guess.

Fig S2 shows analytical and numerical results for the driving pressure in passages with the same total elevation difference $\Delta z = 100m$, length $L = 500m$, constant diameter $D = 2m$, $k = 0.5$, but varying Δz_1 and Δz_2. Longitudinal profiles, therefore, change from an L-shape to a Γ-shape profile as shown on the insert. The temperature difference is $\Delta T = \pm 15°\text{C}$ for both, downdraft and updraft. Relaxation length is calculated with velocity $v = 1m/s$. The velocity in numerically rersults is around 3 m/s, which gives a longer relaxation length and explains lower driving pressure in numerical results.
