# Supplementary material for: How do caves breathe: The airflow patterns in karst underground
Source: PLoS One. 2023 Apr 3;18(4):e0283767. doi: 10.1371/journal.pone.0283767 (PMC10069778; doi:10.1371/journal.pone.0283767)
Supplement: Attachment — Submitted filename: Response to reviewers.docx [file pone.0283767.s002.docx]

**Response to reviewers**

I sincerely thank to both reviewers for recognising the importance of this work and suggestions for improvement. Below find a list of comments and my answers and reactions to them. All line number given correspond to numbering in the file with marked changes (Gabrovsek-plos-tracked.pdf)

**REVIEWER 1:**

**Comment:** The reviewer reminds on the work of Lismonde (2001) on V-shaped passage. I have (with my limited French and electronic translation) read the paper, which recognises the instability in the V-shaped passage. In my work I elaborate the idea into a numerical result.
**Response:** I have now included citation and I mention the findings in the text. **Line 353 in the tracked version, Reference 27.**

**Comment:**  Paragraph "Driving pressure of the chimney effect..." (no line numbering here): Starting from the inlet E1 (not E2!) at the elevation z1 and pressure p1...
**Response:** Corrected. **Line 101 in tracked version.**

**Comment:**  124 : author mentions "most of our observation sites...", whereas only 2 sites are documented before. Only at the final end of the paper, he discusses other sites. Be clearer, and make the difference between qualitative field observations and studied sites with quantitative records.
**Response:** Corrected: **Line 145 to 148 in tracked version.**

**Comment:**  293: In following scenarios the diameter in the vicinity...
**Response:** Corrected. **Line 322 in tracked version.**

**Comment:** 384: The results are based on approximations and assumptions that are (in < remove) not always valid
**Response:** Corrected. **Line 423 in tracked version.**

**REVIEWER #2: 
Comment:** Lines 103-123: It seems to me that the described example is mostly based on the study published by Kukuljan et al. 2021, which is not noted in this section (otherwise monitoring setup should be described in more detail).
**Response:** I added the text stating that the measurement system is described in Kukuljan (2021): **Lines 142-144 in tracked version.**

**Comment:** Fig. 13, Lines 313-322: According to the figure (and description within the text) it seems that in the case of colder outside air (-5°C), the air circulation in the cave is directed from the higher entrance (z+δz) to the lower one (downdraft). This is in contrast to the previous examples (updraft during the period of colder outside air), and also contrary to the described example of the "Kačna" Cave (Figure 15), where during the cold season the cold air enters through the lower (main) entrance of the cave. So, it looks that there is a mistake on the Figure 13 regarding airflow direction?
**Response:**  In a V-shaped passage it is the imbalance (or density difference) between the both limbs of the passage that triggers the airflow. To trigger the initial airflow, which drives the system into a feed-back loop, here a minimal elevation difference δz between both entrances was used. However, as the reviewer recognised this would cause flow from lower to higher entrance, which is the case. So the direction on Figure 13a is wrongly marked and has been corrected**.**
The topography above Kačna jama is drawn schematically. The actual airways where air returns to the surface are not really known; but they must exist. Also not known is the position of oulets (blow holes) at the surface. The initial imbalance may ibe caused by the small altitude difference or by other pertrubations, such as surface winds. I have redrawn Figure 15 and changed the caption to avoid confusion.
**See Fig 15 and caption between lines 386 and 387 in tracked version.**

**Comment:** Line 323: Luetcher et al (2008) also noted cold trap behavior of u-shaped cave.
**Response:** Citation to Luetcher et al. (2008) is added **in line 392 (tracked version), where importnce of cold traps for the formation of ice caves is mentioned.**

**Comment:** Figure 15: Short explanation for the varying colors of the arrows indicating airflow direction could be added.
**Response:** Added. **See caption of Fig 15, between lines 386 and 387 in tracked version.**

**Comment:** Lines 368-380, Figure 16: Example of "Snežnik" Cave is interesting but slightly confusing: if the mentioned entrance is at the highest point of the massif, the formation of ice in it should indicate a downdraft during the cold season, which contradicts the previous considerations? Also, the model shown in Figure 16 includes two inlets approximately at the same height, while the Snežnik example probably has a "snow covered" inlet significantly above the other one? Not sure if the model would show a similar results for a configuration more suitable for Snežnik example.
There is a recently published study of monitoring results from a very deep cave with extensive ice deposits (Velebit Mt., Dinaric Karst), which interprets the functioning of the cave as a cold-trap that causes a negative temperature anomaly within the karst massif (in accordance to your results and considerations). **Response:** Maybe giving Snežnik cave as an example might be a bit of a speculation. However, we know only one entrance leading to 630 m deep system of shafts with. We have observed dowdraft in the cave in all seasons and the presence of ice through most of the seasons between depths of 100 m and 200 m. The outlet of the air could be higher, but according to local topography it is hard to imagine that it is significantly higher; maybe 100 m, which is, taking into account that the downdraft is still present at the depth of 600 m, not very significant. However, idea that the presence of ice in the known limb keeps the air density in the limb high and sustaind dowdraft in all seasons.I am familiar with research on Velebit mountain. I am not very sure which publication the reviewer has in mind. However, I believe that the model could be used to interprete many »unusual« situations.

**Comment:** Conclusion section: I propose to additionally include brief conclusions related to the influence of object morphology (L-shaped, V-shaped, varying diameter) on seasonal airflow patterns.

**Response: Done, see lines 416-421 in tracked version.**
